# The Reliability and Validity of the Japanese Version of the Modified Dental Anxiety Scale among Dental Outpatients

**DOI:** 10.1155/2020/8734946

**Published:** 2020-05-01

**Authors:** Mika Ogawa, Teppei Sago, Hirokazu Furukawa

**Affiliations:** ^1^Division of Dental Anesthesiology, Kyushu Dental University, Fukuoka, Japan; ^2^Section of Anesthesiology, Department of Diagnostics & General Care, Fukuoka Dental College, Fukuoka, Japan; ^3^Department of Clinical Psychology, Naruto University of Education, Tokushima, Japan

## Abstract

**Introduction:**

A careful assessment of dental anxiety is necessary for its management. The Modified Dental Anxiety Scale (MDAS) is one of the most commonly used questionnaires to measure dental anxiety in the world. The reliability and validity of the Japanese version of MDAS have been demonstrated using undergraduates and a few patients with dental anxiety. The aim of the present study was to examine the reliability and validity of the Japanese version of the MDAS using a wide range of age samples in dental clinics.

**Methods:**

A total of 275 outpatients (145 men and 130 women; 21–87 years) from two dental clinics participated in the present study. Dental anxiety was assessed using the Japanese version of the MDAS and the Dental Fear Survey (DFS). The psychometric evaluation included exploratory factor analysis, and Cronbach's *α* was used to evaluate for internal consistency. Criterion validity was assessed by correlating the MDAS and DFS scores using Spearman's correlation coefficient. validity was evaluated by examining related factors' differences in the MDAS score (e.g., sex and negative dental experiences).

**Results:**

Six patients (2.2%) reported high levels of dental anxiety (MDAS score ≥ 19). The internal consistency of the MDAS score was high (Cronbach's *α* = 0.88). Dental anxiety was significantly higher among women (*P*=0.007), in patients with previous negative dental experiences (*P* < 0.001), and among those with lower frequencies of dental visits (*P* < 0.001). The MDAS score was significant and related to age (*r* = 0.48) and the DFS score (*r* = 0.87). Factor analysis revealed all items measured only one construct.

**Conclusions:**

The Japanese version of the MDAS score was found to be a reliable and valid measure of dental anxiety among dental outpatients. It could be useful for the Japanese dental practitioner to measure dental anxiety in a clinical setting.

## 1. Introduction

Dental fear and/or anxiety can cause patients to avoid dental treatment and consequently result in poor oral health that is related to quality of life [1–4]. Moreover, treating a highly fearful patient, including a child, is a negative experience for dental practitioners [5]. Thus, it is imperative to address dental fear.

It has been suggested that a careful assessment of dental anxiety is necessary for its management [6]. Even after establishing numerous measures of dental anxiety, only 20% of dental practitioners utilize measures of dental anxiety that have been developed for use with adults [7]. In Japan, there is no available evidence on how practitioners use these tools. The Japanese version of the Dental Fear Survey (DFS) [8] and the short version of the Dental Anxiety Inventory [9] have already been published, and they have been found to be reliable and valid.

The Modified Dental Anxiety Scale (MDAS) was developed in 1995 as an improvement on Corah's Dental Anxiety Scale (CDAS), and it has been translated into 22 different languages [10]. The CDAS comprises four dental items, but it does not include an item that assesses the patient's fear of local anesthetic injections [11] even though local anesthetic injections are one of the strongest stimuli to evoke dental anxiety [12]. The MDAS comprises five items, including the Dental Anxiety Scale and an item for local anesthetic injection. In addition, patients with a high level of dental anxiety could be distinguished from nonanxious patients using a cutoff score of the MDAS [10]. Therefore, the MDAS has advantages when compared with other questionnaires because of its quick administration and screening of patients with dental anxiety. It is therefore commonly used in dental clinics and epidemiological surveys.

The reliability and validity of the Japanese version of MDAS have been demonstrated using two samples: individuals with high levels of dental anxiety (*N* = 9) and undergraduate students (*N* = 208) [13]. However, the previous study was not conducted in a clinical setting (e.g., a dental clinic) and did not use a wide range of age samples even though numerous studies reported MDAS score related to age [14–21]. The evaluation of validity is focused on inferencing the suitability of subjects who have produced test scores rather than the properties of the tests [22]. The validation process is considered to be integrated and the subjective process based on different sources of evidence (e.g., theory and empirical evidences) [23]. Therefore, we attempted to indicate further evidence for some psychometrics of the Japanese version of the MDAS using a clinical sample with a wide range of ages.

The aim of the present study was to examine the reliability and validity of the Japanese version of the MDAS among dental outpatients in Japan and to compare the prevalence of high levels of dental anxiety in Japan with results in other countries.

## 2. Methods

### 2.1. Participants

In the present cross-sectional study, participants were recruited from two general dental clinics in Kitakyushu and Shimonoseki in October 2018. This study performed a consecutive sampling, and the sample inclusion criteria were as follows: over 20 years of age, Japanese as a mother tongue, and those who could understand the questionnaire and write an answer. The adequate sample size for a factor analysis is controversial but roughly evaluated as follows: 200-fair; 300-good; and 500-very good [24]. Out of the 300 questionnaires that were distributed to the patients, eight patients refused to participate in the study, and 292 were completed and deemed to be eligible for inclusion in the study. After the participants provided informed consent, they responded to a self-administered questionnaire while they sat in the waiting room of the dental clinic. The present study was approved by the Ethics Committee of Kyushu Dental University (No. 18–24).

### 2.2. Measurements

#### 2.2.1. MDAS

The MDAS is a 5-item questionnaire that assesses the respondents' emotional reactions to 5 situations: planning to visit a dental clinic the following day, waiting in the waiting room of a dental clinic, having one's teeth drilled, having one's teeth scaled, and receiving local anesthetic injections [10]. Responses are recorded on a 5-point Likert-type scale from “not anxious” to “extremely anxious.” Total scores could range from 5 to 25, with higher scores indicative of greater dental fear. A cutoff score of ≥19 was used to identify individuals with high levels of dental anxiety [10].

The Japanese version of the MDAS was translated, and the cross-cultural adaptation was assessed using a “forward-backward blind translation” process that was delineated by Furukawa and Hosaka. The first translation was performed by the third author and a bilingual dentist who specialized in dental anxiety, and a bilingual psychologist revised the first translation and compared the Japanese version to the original. The back translation was approved by the developers of the original version of the MDAS [13].

#### 2.2.2. DFS

The DFS is a 20-item self-report measure that is commonly used to assess the behavioral and physiological responses that accompany dental anxiety or fear [12]. Responses are recorded on a 5-point Likert-type scale, and the total score can range from 20 to 100; higher scores are indicative of greater dental fear. The questionnaire comprises three dimensions: avoidance (e.g., delay making an appointment), physiological arousal (e.g., muscles become tense and breathing rate increases), and fear of specific situations (e.g., seeing the needle of an anesthetic injection and feeling the drill against one's tooth). The DFS was developed in 1973 by Kleinknecht et al. [12], and it has been translated into numerous languages [8, 25–28]. The Japanese version of the DFS is found to be of high reliability using Cronbach's alpha ranging from 0.94 to 0.96 [8].

Demographic information (e.g., sex and age) as well as patients' dental history (e.g., frequency of dentist visits and previous negative dental experiences) were also recorded.

### 2.3. Statistical Analyses

Because the distribution of the MDAS and DFS scores was skewed (few participants had high levels of dental fear), nonparametric tests were used. Spearman's correlation analysis was used to examine the relationship between the scores yielded by the MDAS and DFS. The Mann–Whitney *U* test and the Kruskal–Wallis test were used to examine demographic differences (e.g., sex and age) in the MDAS scores. Internal reliability coefficients of the components in the scale were calculated with Cronbach *α*. Factor analysis was used to examine whether the MDAS is undergirded by a singular dimension. Exploratory factor analysis with a maximum likelihood and promax rotation was performed. Kaiser-Meyer-Olkin test (KMO) was performed for sampling adequacy of the factor analysis. All the statistical analyses were conducted using version 24 of SPSS (SPSS, IBM Japan, Tokyo).

## 3. Results

Of the 292 participants who were initially included in the study sample, 17 were excluded because they had submitted incomplete questionnaires. The questionnaire response rate was 94.2% (i.e., 275/292). The descriptive statistics for the study variables are presented in Table 1.

Of the 275 respondents, 145 were men (52.7%) and 130 were women (47.3%). The mean age of the patients was 50.05 years (standard deviation (*SD*) = 17.59; range = 21–87 years). The mean of the total MDAS score was 10.89 (*SD* = 3.75; range = 5–22). Overall, six patients (2.2%) reported high levels of dental fear (i.e., MDAS score ≥ 19).

### 3.1. Internal Consistency Reliability

In the present study, Cronbach's alpha of the MDAS was 0.88.

### 3.2. Criterion Validity

Table 1 shows the descriptive statistics for the scores yielded by the MDAS and DFS. A significant and strong correlation was observed between the total scores of the MDAS and DFS (*r* = 0.87, *P* < 0.01). A significant correlation was also observed between the MDAS total score and the three dimensions of the DFS, namely, avoidance (*r* = 0.83, *P* < 0.01, physiological arousal (*r* = 0.81, *P* < 0.01), and the fear of specific situations (*r* = 0.87, *P* < 0.01).

### 3.3. Construct Validity

Table 2 shows the mean MDAS scores that were obtained by the groups that differed in demographic characteristics and dental anxiety-related variables.

*Age*. Younger age groups obtained significantly higher mean MDAS scores than the older age groups (*P* < 0.001). Age and MDAS scores have a positive correlation (*r*=0.48, *P* < 0.01).

*Sex*. Women obtained significantly higher mean MDAS scores than men (*P*=0.009).

*Dental Attendance Pattern*. Participants who regularly visited their dentists obtained significantly lower mean MDAS scores than those who visited their dentist irregularly (*P* < 0.001).

*Negative Experiences*. Participants who reported previous negative dental experiences obtained significantly higher mean MDAS scores than those who did not report previous negative dental experiences (*P* < 0.001).

### 3.4. Factor Analysis

A factor analysis of the responses that were provided to the MDAS extracted one factor that had an eigenvalue that was greater than 1 (eigenvalue for the first factor = 3.43); the KMO measure of sampling adequacy was 0.82. KMO values between 0.8 and 1 indicated that the sampling was adequate [29]. The extracted factor explained 68.6% of the item variance. The factor loadings of the items, all of which were above the accepted threshold (>0.5) [30], are presented in Table 3.

## 4. Discussion

The Japanese version of the MDAS demonstrated high levels of internal consistency, criterion validity, and construct validity among outpatients of the general dental clinics. A factor analysis of the Japanese version of the MDAS revealed that all the scale items measure only one construct.

The Japanese translation of the MDAS demonstrated strong internal consistency reliability. Reliability refers to the extent to which the scores that are yielded by a questionnaire can be replicated [31]. There are three types of reliability: equivalence, stability, and internal consistency. Stability is often assessed through a test-retest procedure that administers the same questionnaire to the same individuals under the same conditions [31]. In the present study, all the participants were outpatients, and their levels of dental anxiety could be changed due to the dental treatment; therefore, test-retest reliability was considered not to be appropriate in the present study. In the present study, internal consistency was measured using Cronbach's alpha. Internal consistency indicates the extent to which items in the test are measuring the same thing [31]. The Japanese version of the MDAS demonstrated a high Cronbach's alpha value. Cronbach's alpha value of the Japanese version was relatively similar to the Finnish and Arabic version [32], the Italian version showed higher [16], and the Nepali [33] and Malay [21] versions showed lower values than the Japanese version among dental patients (shown in Table 4).

The Japanese version of the MDAS demonstrated strong criterion-related validity among dental outpatients. Validity refers to the number of systematic errors that a questionnaire generates [31]. Criterion-related validity refers to how well a given questionnaire relates to an external criterion. Similar to the Turkish version of the MDAS, the scores that were yielded by the Japanese version of the MDAS were significantly correlated with the DFS scores [34].

The Japanese version of the MDAS scores also demonstrated strong construct validity. Construct validity refers to the extent to which a questionnaire measures the trait or theoretical construct that it is intended to measure [31]. Therefore, groups that were known to differ in their levels of dental anxiety (e.g., age, sex, dental attendance patterns, and negative experiences) were compared with the scores that were yielded by the Japanese version of the MDAS in the present study.

The present results suggest that mean MDAS scores decrease as age increases in agreement with many studies in UK, Italian, Arabic, Indian (Kannada and Tamil), Chinese, and Malay populations [15–21], whereas a study in Turkey reported that MDAS scores increased with age [34]. In the present study, female patients reported higher levels of dental anxiety than male patients. This result is also consistent with earlier findings in UK [15], Italian [16], Greek [35], Arabic [17], Malay [21], Turkish [34, 36], and Chinese [20] populations. Sex differences are evident in the prevalence rates of specific fears and phobias [37]. On the contrary, a study reported that there was no sex difference among Nepali patients [33]. Ethnic and cultural differences could attribute these differences among demographic variables [33].

The MDAS score was significantly higher among patients who had previous negative dental experiences and patients who had a lower frequency of dental visits. Previous studies also reported negative dental experiences were also associated with dental anxiety [18, 21, 34, 38]. Furthermore, patients with higher levels of dental anxiety visited their dentists less frequently [15, 17, 20, 21]. These findings are consistent with the “vicious cycle” of dental anxiety [39]. This cycle indicates that a negative dental experience arouses dental anxiety and results in delayed dental attendance.

In the present study, 2.2% of the outpatients reported phobic levels of dental anxiety (i.e., scores that were ≥19 on the MDAS) [10]. The prevalence in the present study was relatively similar to that which has been observed among dental patients in Nepal [33], India [18], Finland (Helsinki) [32], and Malaysia [21]. On the contrary, the results of some studies [16, 32] reported levels of dental anxiety that were higher than what were observed in the present study (see Table 4). The differences in prevalence rates may be attributable to differences in the sampling technique. Specifically, patients who are receiving emergency care [32] or undergoing oral surgery [16] may report higher levels of dental anxiety than patients who are visiting their general dental practitioner.

This study has some limitations. First, the present sample was recruited from only two dental clinics using nonprobabilistic sampling; thus, there is a possibility of a sampling bias. Therefore, the prevalence rate of dental anxiety in the present study (2.2%) cannot be generalized to the entire Japanese population. To avoid the bias, additional research using the nationwide random sample is needed. Second, participants completed a questionnaire while awaiting dental treatment in the clinic's waiting room, and therefore, social desirability bias could occur. Third, limited demographic variables (only age and sex) were measured in the present study. The study did not evaluate educational and income level in terms of protecting individual's privacy. However, previous studies reported that people who have a lower socioeconomic background correlated with higher levels of dental anxiety because low income may result in delayed dental attendance and symptom-driven treatment [19, 21, 39, 40]. The association between other demographic variables (e.g., education level, income, and occupation) and dental anxiety among the Japanese population is needed in future research.

## 5. Conclusions

The Japanese version of the MDAS demonstrated good psychometric properties among dental outpatients. Thus, this questionnaire can be used to quantify the dental anxiety of Japanese patients in clinical settings.

## Figures and Tables

**Table 1:** Descriptive statistics for the MDAS and DFS scores.

Variable	Mean	*SD*
MDAS	10.89	3.75
DFS	38.54	13.30
Avoidance	13.92	5.15
Physiological arousal	8.98	3.11
Fear of specific situations	15.64	5.84

MDAS = Modified Dental Anxiety Scale. DFS = Dental Fear Survey. *SD* = standard deviation.

**Table 2:** Significance of the difference in MDAS scores between groups that differed in demographics and dental anxiety-related variables.

Variable	*N*	%	MDAS		*P*
			Mean	*SD*	
Sex					
Male	145	52.7	10.01	3.22	0.009^*∗*^
Female	130	47.3	11.88	4.06	
Age (years)					
20–39	99	36.0	12.80	3.40	<0.001^†^
40–59	90	32.7	10.60	3.48	
>60	86	31.3	9.01	3.37	
Frequency of dental visits					
Regular	54	19.6	8.72	3.04	<0.001^†^
Occasional	122	44.4	11.28	3.56	
Only when there is pain/trouble	99	36.0	11.61	3.93	
Previous negative dental experience					
No	191	69.5	10.20	3.68	<0.001^*∗*^
Yes	84	30.5	12.48	3.44	

^*∗*^Mann–Whitney *U* tests; ^†^Kruskal–Wallis tests. MDAS = Modified Dental Anxiety Scale. *SD* = standard deviation.

**Table 3:** Factor loading of the MDAS items.

Items	Mean	SD	Factor loading
MDAS item 1	1.99	0.83	0.88
MDAS item 2	2.09	0.90	0.86
MDAS item 3	2.49	0.98	0.90
MDAS item 4	1.50	0.72	0.64
MDAS item 5	2.83	1.08	0.84

MDAS = Modified Dental Anxiety Scale. *SD* = standard deviation.

**Table 4:** Comparison of the prevalence rates of high levels of dental anxiety and mean MDAS score between dental patients of different countries and the present Japanese sample.

Nation	*N*	MDAS score ≥19	Mean MDAS	*SD*	Age (years)	Internal consistency	Dental setting
United Kingdom (original)	848	NA	10.79	4.63	20–60 or older	NA	General dental practitioners
Nepal	150	2.0%	12.29	3.03	16–42	0.78	Department of orthodontics
Japan (present study)	275	2.2%	10.89	3.75	21–87	0.88	General dental practitioners
India	146	2.7%	9.68	3.75	18–70	0.84	Dental college hospital
Finland (Helsinki)	200	3.0%	9.44	3.91	16–65 or older	0.89	Dental hospital
Malaysia	455	3.5%	10.73	4.03	16–56 or older	0.85	University hospital, outpatients of dental clinics
United Arab Emirates	200	6.0%	10.90	4.28	16–65 or older	0.86	Dental hospital for patients who require emergency care
Finland (Jyväskylä)	194	8.8%	8.80	4.65	16–65 or older	0.88	Dental hospital for patients who require emergency care
Northern Ireland	200	19.5%	19.50	5.98	16–65 or older	0.90	General dental service of the National Health Service
Italy	230	19.6%	12.68	5.07	14–88	0.92	Patients undergoing oral surgery

MDAS = Modified Dental Anxiety Scale. *SD* = standard deviation. NA = not available.

## Data Availability

The data used to support the findings of this study are available from the corresponding author upon request.
